# Attentional bias assessed by a facial expression cuing paradigm in infants

**DOI:** 10.1038/s41598-018-36806-1

**Published:** 2019-01-11

**Authors:** Atsuko Nakagawa, Masune Sukigara

**Affiliations:** Graduate School of Humanities and Social Sciences, Nagoya City University, 1 Yamanohata, Mizuho-cho, Mizuho-ku, Nagoya, 467-8501 Japan

## Abstract

To disambiguate infants’ attentional bias towards fearful facial expressions, we applied a facial expression cueing paradigm to 36 6-month-old and 33 12-month-old infants, with 21 infants taking part at both ages. Infants made saccades towards a peripheral target preceded by a happy, fearful, or neutral cue directing their attention to the target location (congruent) or the wrong location (incongruent). The results show that infants were faster to respond when shown a fearful (vs. happy) face as a congruent cue, which is consistent with previous studies referring to fearful vigilance, while an incongruent fearful cue reduces attention shifts to the target on the opposite side of the monitor to a greater extent than an incongruent happy cue at 12 months, implying that a fearful facial expression prolongs attentional disengagement or is associated with a greater narrowing of attention. Additionally, the latencies of 6-month-olds were significantly faster than those of 12-month-olds in a congruent condition. The relationship between attentional bias and temperamental disposition was examined using the Infant Behavior Questionnaire–Revised. High temperamental orienting scores partly correlated with attentional bias at 12 months. The contributions of attentional brain networks to socio-cognitive and emotional development are also discussed.

## Introduction

Attentional bias related to threat or fearfulness has been reported as either a rapid orientation towards fearful stimuli or slow disengagement from fearful stimuli^1^. While these biases are typically detected as behavioural reaction time responses in adults and older children, they have been shown to be present during infancy (for reviews, see^2,3^). Using eye-tracking technology, 8- to 14-month-olds were found to turn more quickly to threatening than to non-threatening stimuli in a visual search task in which they were presented with various stimuli^4^. This appears to constitute an orienting advantage towards threat. The influence of a fearful facial expression in infants was also reported as attention disengagement in 7-month-olds when a central face is removed in gap trials prior to the appearance of the peripheral target, while in overlap trials, the central face stimulus remains present throughout the trial^5,6^. A decreased probability of attention being disengaged from fearful faces and greater and longer-lasting heart rate deceleration in response to fearful compared to non-fearful expressions throughout the task^7^ indicate a difficulty in disengagement from threatening stimuli.

Although evidence has gradually accumulated, there has been little work on attentional bias for threat in infancy. Referring to previous studies^8,9^, Peltola *et al*.^7^ show that functional maturation of the neural circuitry responsible for directing attention to emotionally significant signals may be linked to pronounced attentiveness to fearful faces at 7 and 9 months. An emotion-processing network (the amygdala and the orbitofrontal cortex) connecting to visual and attention-related systems (face-sensitive regions in the fusiform gyrus and the posterior superior temporal sulcus) may be functionally matured in the second half of the first year of life^3^. Moreover, Peltola *et al*.^10^ indicate that 7-month-old infants’ attentional bias towards fearful faces is associated with attachment formation (secure attachment) at 14 months, applying the overlap paradigm and the strange situation procedure (SSP; Ainsworth *et al*.^11^) in a longitudinal study.

On the one hand, according to the brain-network theory of attention (Posner and colleagues)^12,13^, attentional bias towards fearful faces in infancy is related to the attention-orienting brain network, which includes the superior parietal cortex, the temporal parietal junction, and the frontal eye fields. Subcortical areas, including the pulvinar and superior colliculus, are also involved. The function of the orienting brain network is to align attention with the source of sensory signals in order to prioritize it, which plays a part mostly in initial orientation and disengagement. This orienting network is thought to play the main role in regulation in infancy. Later in childhood, the executive attention network, including the anterior cingulate, insula, and areas of the basal ganglia, becomes dominant as a mechanism of self-regulation. Executive attention involves the voluntarily regulating of behaviour and is responsible for resolving conflicts between dominant but inappropriate responses and non-dominant courses of actions (e.g., inhibiting immediate rewards in order to accomplish future goals, etc.). Attentional engagement, or sustained attention, may also be associated with the function of executive attention.

The efficiency of orienting attention in infancy and executive attention from toddlerhood and beyond as measured by parent- and self-reports in temperamental behaviour questionnaires are reported to be significantly positively correlated^14^. The efficiency of executive attention is called “effortful control”^12,13^. Recent work^15^ shows that the development of effortful control is influenced by parent-child attachment security. Combined with the findings of Peltola *et al*.^10^, attention-orienting brain network in the first year may be associated with the efficiency of executive attention or effortful control mediated through attachment formation. That is, infants with higher orienting/regulation scores may show stronger attentional bias to the threat. This may be consistent with the indication that in the overlap paradigm with fearful expression, 12-month-old infants with higher orienting/regulation scores focused longer on the threatful stimuli^16^. It is also suggested that the orienting attention system in infancy responds differently to non-threatening and threatening (fearful) stimuli^17^.

**Figure 1:**
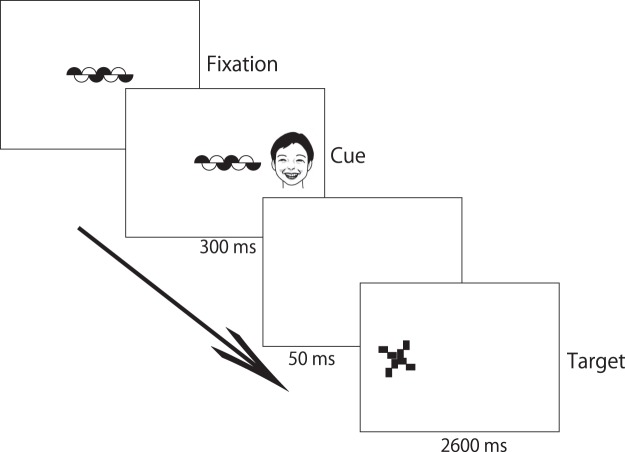
Schematic illustrations of the presentation of cue and target stimuli in incongruent trials (Peripheral cues were pictures of individuals with fearful, neutral, or happy facial expressions).

So far, the overlap paradigm used in previous studies^5–7,10,16^ has failed to disentangle whether slower latencies for disengaging or fewer attention shifts from fearful facial expressions compared to neutral and happy facial expressions are explained by increased attentional engagement or increased difficulty in disengaging by fearful stimuli or both. Thus, in the present study, a facial expression cuing paradigm was applied to infants (Fig. 1). This task, which was adapted from Posner *et al*.^18^, was introduced to investigate the ability to disengage attention as separate from other components of attention in adults^19^. In the original spatial cuing paradigm^18^, participants are required to detect a target (e.g., an asterisk) that may appear to the left or the right of the fixation point. The peripheral target is preceded by a cue (e.g., a flashing light) directing their attention to the target location. The cue correctly predicts the target location (i.e., a congruent cue) in 80% of trials, whereas the target appears on the opposite side of the cue (i.e., an incongruent cue) in 20% of trials. That is, an exogenous cue causes covert orienting attention to the cued location, generating faster latencies in congruent trials and slower latencies in incongruent trials and indicating the acceleration of initial attentional engagement and the prolongation of attentional disengagement, respectively.

In recent research using a spatial cuing paradigm in adults^19^, the authors applied anti-expressions as one of a number of control stimuli, which are emotionally neutral, controlling for visual features specific to emotional facial expressions relative to neutral expressions. They also asked participants to evaluate each stimulus in terms of their subjective emotional experience. As a result, normal emotional facial expressions of anger and happiness decreased reaction times for engagement of attention in a valid condition and increased reaction times for disengagement of attention in an invalid condition, compared with anti-expressions. Moreover, higher subjective arousal ratings were related to shorter reaction times in valid trials and to longer reaction times in invalid trials. The authors conclude that emotional signals but not visual effects on the perception of emotional facial expressions accelerate attentional engagement and prolong attentional disengagement.

Although prior studies used the facial expression cuing paradigm with older children^20^ or adults recruited from the community or undergraduate samples^21,22^, the present study is a novel downward application with respect to age. Here, infants made saccades towards a peripheral target preceded by an exogenous facial expression cue (happy, fearful, or neutral) while directing their attention to the target location (congruent cue) or the wrong location (incongruent cue). Indication of where the target will appear is the location of the facial expression cue, not the facial expression itself. We examined 6- and 12-month-olds longitudinally. Infants recognize and differentiate between emotional facial expressions by 7 months of age^9^ and ERP studies have shown that Nc (Negative Central, about 450–550 ms after stimulus onset) is larger in amplitude when looking at fearful than at happy expressions^23,24^, which is thought to reflect a general arousal/attention system in the brain^25^. Therefore, we hypothesized that compared with neutral or happy stimuli, fearful stimuli should delay responses to subsequent targets in an incongruent condition and quicken responses to subsequent targets in a congruent condition in 12-month-olds but not in 6-month-olds.

In addition, we examined the relationship between measures of attentional bias and temperamental disposition by administering the Infant Behavior Questionnaire–Revised (IBQ-R)^26^. The IBQ-R was developed based on a definition of temperament as consisting of individual differences in reactivity (e.g., how easily our emotions, motor activity, and attention are aroused) and self-regulation (processes that act on reactive tendencies, such as attention). The score for orienting/regulation is distinct from the two broad temperament factors of surgency and negative affectivity. Because a significant correlation between maternal reports of negative affect and attention to threat at 12 months^16^ have been reported, we investigated how attentional bias is related to temperament at each age, using negative affectivity or fear scores and orienting/regulation scores on the IBQ-R instrument.

## Results

To treat the uneven distribution of the dependent variables, we applied a log linear transformation to latencies and an arcsine transformation to response probabilities. Response probabilities and latencies for each condition are presented in Table 1.

**Table 1:** Number of responses, mean probabilities and latencies for responses towards targets in congruent (a) and incongruent (b) experimental conditions and age (following arcsine and log transformations, respectively).

Condition	Expression	Number of responses	Latency	Probability
**(a) Congruent**				
6 months (N = 16)	Neutral	8.00 (2.30)	415.03 (101.29)	0.95 (0.11)
	Fearful	7.94 (3.15)	381.56 (100.91)	0.95 (0.09)
	Happy	7.56 (3.07)	401.28 (92.09)	0.91 (0.12)
12 months (N = 16)	Neutral	7.75 (3.08)	495.49 (84.61)	0.95 (0.07)
	Fearful	7.56 (3.24)	469.57 (109.83)	0.90 (0.13)
	Happy	7.62 (2.84)	540.39 (134.63)	0.94 (0.07)
**(b) Incongruent**				
6 months (N = 14)	Neutral	3.14 (0.66)	883.13 (89.84)	0.95 (0.10)
	Fearful	2.86 (0.86)	929.66 (81.36)	0.95 (0.12)
	Happy	2.86 (0.77)	1013.59 (106.60)	0.97 (0.08)
12 months (N = 20)	Neutral	2.85 (0.81)	836.51 (105.46)	0.91 (0.13)
	Fearful	2.70 (0.65)	840.34 (131.86)	0.90 (0.14)
	Happy	3.05 (0.88)	877.79 (125.45)	1. 00 (0.00)

Latencies are in milliseconds. Standard deviations are given in parentheses.

Data are based on participants responding during more than two trials per condition.

Data in the congruent condition are longitudinal.

A 3 (facial cues) × 2 (ages) repeated measures ANOVA was conducted on latencies for the congruent condition based on longitudinal data from 16 infants. As a result, the main effects of both age and facial cues were significant (*F* (1, 15) = 11.95, *p* < 0.01, *ηp*^2^ = 0.43; *F* (2,30) = 4.70, *p* < 0.05, *ηp*^2^ = 0.23). That is, responses at 6 months were significantly faster compared to those at 12 months in the congruent condition. Regarding facial cues, paired comparisons revealed that eye movements towards the location of fearful expressions cue were faster than towards that of happy expressions (*p* = . 055). This interaction was not significant (*F* (2,30) = 1.37, *p* > 0.10). The same analysis was conducted for probability, for which neither the facial nor the age main effect was significant (*F* (1, 15) = 0.30, *F* (2,30) = 0.61, *ps* > 0.10). This interaction was not significant (*F* (2,30) = 1.43, *p* > 0.10).

In the incongruent condition, latencies at 6 months (*N* = 14) and 12 months (*N* = 20) were analysed through a one-factor ANOVA because these included the longitudinal data of 5 participants. While we did not find a main effect of facial expression at 12 months (*F* (2, 38) = 1.41, *p* > 0.10), that effect was significant at 6 months (*F* (2, 26) = 8.71, *p* < 0.01, *ηp*^2^ = 0.40). That is, eye movements when shown the happy cue were slower than those when shown the neutral facial cue in a incongruent condition (*p* < 0.05).

A one-factor (facial expressions) ANOVA was conducted on probability for incongruent conditions at each age. There were no significant main effects of facial expressions at 6 months (*F*(2, 26) = 0.22, *p* > 0.10), whereas we found significant main effects of facial expressions at 12 months (*F*(2,38) = 4.10, *p* < 0.05, *ηp*^2^ = 0.17). Paired comparisons revealed that probability decreased significantly when showing the fearful or neutral cue compared to showing the happy cue in the incongruent condition (*p* < 0.05).

To confirm infants’ engagement to the cue in the incongruent condition, we examined infants’ initial gaze shift to the location of face cues prior to the target on the opposite side, some of which moved from the cued location to the target. These responses showed overt attention in the incongruent condition. Table 2 presents the probability of these responses, or the number of responses to the cued location divided by the number of fixated responses in each condition. A one-factor (facial expressions) ANOVA was conducted at each age. Neither the main effect at 6 months nor that at 12 months was significant (*F*(2, 26) = 1.92, *p* = 0.166; *F*(2, 38) = 1.78, *p* = 0.182).

**Table 2:** Mean probabilities for responses towards the cue in the incongruent condition of each age (following arcsine transformations).

Incongruent Condition	Expression	Probability
6 months (N = 14)	Neutral	0.70 (0.31)
	Fearful	0.82 (0.23)
	Happy	0.70 (0.26)
12 months (N = 20)	Neutral	0.47 (0.37)
	Fearful	0.55 (0.35)
	Happy	0.61 (0.34)

Standard deviations are given in parentheses.

To address the relationship between attentional bias to threat and temperament, infants who went through more than two trials per cell of three facial expressions in both conditions were analysed at each age (6 months: *N* = 14; 12 months: *N* = 20; see Table 1b). Correlation coefficients were calculated between temperamental score (temperamental orienting/regulation, negative affect, or fear scores) and index for attentional bias (latency in the congruent condition with a fearful face cue, response probability in the incongruent condition with a fearful face cue, and probability of infants’ initial gaze shift to face cues in the incongruent condition). Results show that orienting/regulation scores at 12 months were negatively related to latency in the congruent condition with a fearful face cue (*r* = −0.539, *p* = 0.014) and positively related to the probability of infants’ initial gaze shift to fearful face cues in the incongruent condition (*r* = 0.445, *p* = 0.046). We found no relationship between attentional bias and temperamental negative affect or fear.

## Discussion

This study applied a spatial cuing paradigm to test whether different facial expressions affect attentional orientating and disengagement in 6- and 12-month-old infants. The clearest result is that infants at both ages find it much faster to move to a congruent-cued than to an incongruent-cued stimulus and to move to a target when correctly cued by a fearful face. In contrast, in the incongruent condition, 6-month-olds seem to let their attention be captured not by fearful but by happy expressions. In contrast, 12-month-olds gave significantly more responses to the targets with incongruent happy expression cues compared to those with incongruent fearful or neutral cues. In both ages, infants seem to discriminate facial expressions presented in a peripheral field.

When the target appeared at the same location as the facial cue (congruent condition), latencies for 6-month-olds were significantly faster than for 12-month-olds. This contradicts the view that saccade latency gradually decreases with age (7–42 months old), suggesting developmental improvements in saccade control^27^. One difference in our findings is that our peripheral stimuli were not colourful cartoon characters but facial expressions. There is thus a possibility that the fast face-processing pathway originating in a subcortical route^28^ worked dominantly in our task with 6-month-olds. Infant ERP analysis^29^ indicates that different brain systems are involved in processing the same emotional face depending on developmental changes between 7 and 12 months. As a dissociation between behavioural results and ERP findings has been reported, further research is needed if we are to understand infants’ saccade behaviour.

In the congruent condition, we also found significantly rapid orientation towards the location cued by fearful expressions compared to the location cued by happy expressions. This is indicative of arousal or vigilance responses to a fearful face, suggesting that infants maintain vigilance concerning fearful faces because of their relevance to threat^8^. LoBue *et al*.^30^ indicate that seeing threat stimuli elicits rapid detection of following stimuli given the finding that infants respond more quickly after viewing trials containing an image of an angry face compared to trials containing an image of a happy face.

Given that there was only a very small number of incongruent trials per facial expression (*N* = 4), our results need to be interpreted cautiously. At 12 months, infants gave significantly more responses to the target in the incongruent condition with the happy expression cue than with the fearful or neutral facial cue. A lack of emotional cue effects in overt attention to cued location (Table 2) shows that our participants engage with the emotional cued location in the incongruent condition regardless of facial expressions as cues. This confirms that the reduced saccades to the targets in the incongruent condition with fearful cue are due to difficulty in disengagement. Sawada & Sato^19^ reported that emotional facial expressions prolong attentional disengagement owing to subjective arousal and that infants show higher arousal in response to fearful than to happy expression^23,24^. Although we did not find longer latencies in the incongruent condition with the fearful expression cue, there is a possibility that it may take more than 2950 ms to make a saccade to the side opposite the target in the fearful condition. Another possibility may be that induced fear, which is high in motivational intensity, causes a narrowing of attention towards external information^31^. In other words, happy faces induced positive moods, which favours exploration and outward attention^32^. Further research is needed to examine these possibilities.

In contrast, at 6 months, latencies were significantly longer with the incongruent happy facial cue than with the fearful or neutral facial cue. This may be interpreted as consistent with infants’ affinity with happy faces because infants are exposed to happiness much more frequently than to other emotions^33,34^. The happy face might enhance emotional arousal, which is associated with the attentional capture or prolonged disengagement of attention^19^. It thus appears that bias towards attending to fearful expressions emerges at the point in development when such expressions are most likely to take place in the infant’s environment, namely, around 6 to 7 months, at which age infants start to crawl and actively explore their environment^3^.

We also examined whether infants with high temperamental orienting/regulation scores or who are temperamentally fearful or more negative demonstrated heightened sensitivity to threat (attentional bias) relative to infants not displaying such temperamental tendencies. Inconsistent with Nakagawa and Sukigara^16^, no significant positive correlation was observed between negative affect and attentional bias. However, the finding that 12-months-olds with high temperamental orienting/regulation scores may attend to threat is consistent with our prediction associated with attachment^10^. One reason why we failed to replicate the relationship with a negative affect may be the difference in the task, as the overlap task^16^ may reflect the stimulus valence^19^ (i.e., the quality of the emotion participants felt towards the facial expression, i.e., negative or positive).

Our study has limitations. Because available trials were few in number, the data generated by the present method may not adequately represent infants’ attention. In particular, the number of trials analysed in the incongruent condition was relatively small because we tried to keep an advantageous ratio of congruent over incongruent trials. In addition, given our adjustable experimental conditions, peripheral emotional processing given approximately 18 degrees from central fixation stimuli either to the left or the right may already be underway in 6-month-olds. Moreover, compared to the 300 ms we set, a relatively brief peripheral facial cue may be appropriate. Infant research frequently uses a brief cue at the target location^35^. Although this cue is so brief that infants rarely shift their gaze in response to it, it does make subsequent eye movements to that location possible. This approach may provide a successful method for drawing attention to the cued location in order to test the influence of emotional stimuli.

This study is innovative in that we applied the visual spatial cue paradigm to infants in order to investigate attentional bias towards threat-related signals in infancy. To date, no study has attempted to disambiguate attentional mechanisms affecting infant behaviours in the overlap paradigm, that is, engaging or disengaging. Our findings show that an incongruent fear cue reduces attention shifts to the target on the opposite side, indicating that fear causes a relative narrowing of attention. We also confirmed the relationship between enhancement of fearful arousal and shortened reaction times in a congruent condition. Given that Peltola *et al*.^7^ have shown that attentional bias to fearful faces appears by 7 months of age and dissipates by 11 months, a sensitive period for processing fear-related information may exist, and attention may therefore mediate most infant socialization. Future studies should examine how and when early attentional components come into play and which of these are relevant to future positive or negative outcomes.

## Methods

### Participants

A sample consisting of 36 6-month-olds (192.75 ± 5.70 days; 16 girls) and 33 12-month-olds (376.24 ± 7.33 days; 14 girls) took part in the experiment. They were recruited through a mailing sent to parents registered in a laboratory-based database of caregivers interested in infant research as well as an advertisement placed in paediatric clinics in Nagoya, Japan. Criteria for inclusion were no known birth or other complications, full term (over 37 weeks of gestation), and normal birth weight (2,500–4,000 g). Additional infants enrolled in the study were excluded from the analyses at 6 (*N* = 14) and 12 months (*N* = 8) because of failure to calibrate at the beginning (6 months: *N* = 3; 12 months: *N = *2) or failure to complete the task as a result of fussiness or crying (6 months: *N* = 11; 12 months: *N* = 6). All caregivers were Japanese mothers who gave informed consent on behalf of their infants before the experiment. The study was approved by the Ethics Committee of Nagoya City University (No. 16004) and was conducted in accordance with the ethical standards specified in the 1964 Helsinki Declaration.

### Procedure

#### Facial expression cuing task and eye movement recording

The experiment took place in a semi-dark area where gaze was recorded using a Tobii TX300 corneal-reflection eye tracker. Each participant sat on his or her mother’s lap at a distance of 60 cm from a 23-inch Tobii TX300 eye tracker display at a resolution of 1920 × 1080 pixels. An experimenter positioned outside this area monitored the subject’s eye movements through a high-angle CCD near-infrared video camera (ELMO CN43H) set in front of the participant, with images of the stimuli being presented superimposed synchronously, using a digital image processor (Roland V-4EX). The experimenter controlled stimulus presentation using E-Prime software and the synthesized moving pictures were recorded (SONY DSR-11) for off-line video coding.

Prior to the start of the study, a five-point calibration procedure was conducted. Following calibration, each trial was initiated by the experimenter pressing a key, and a central fixation stimulus was presented on a black background. Once the infant fixated on the central stimulus, the experimenter pressed a key that triggered the presentation of the facial expression stimulus (i.e., a happy, fearful, or neutral face) as a peripheral cue. This cue was presented for 300 ms at a distance of 18 degrees from the central fixation point either to the left or the right, during which the central fixation remained present. Following 50 ms of blank display, peripheral targets were presented for 2,600 ms either to the left or the right (Fig. 1).

The experiment consisted of 48 trials in which the first 12 trials were given only in the congruent condition to facilitate covert attention to cue location. In the congruent trials, the target was presented in the area indicated by the location of the facial expression cue. In the incongruent trials, the target was presented on the side opposite that indicated by cue location. Following these 12 trials, 36 trials were conducted, consisting of 24 congruent and 12 incongruent trials. Whether the cue appeared to the right or the left of the fixation was determined by a pseudo-random schedule balanced within each set of 12 trials constructed as 4 trials each consisting of 3 facial expression cues. Thus, following the first 12 congruent trials, each set of 12 trials included 4 incongruent trials, with the cues presented equally to the right or the left of the fixation stimuli. A constraint was that the same facial expression cues or the peripheral stimuli on the same side should be presented no more than 3 times in a row.

#### Stimuli

The central fixation stimuli and peripheral targets consisted of brightly-coloured abstract figures animated and subtended at a visual angle of 6 degrees. Each fixation stimulus was initially accompanied by a sound lasting about 500 ms. The sounds consisted of a three-fold repetition of various computer-generated sounds (such as a beep, chime, or bell). The peripheral facial cues used were taken from the ATR Facial Expression Image Database (DB99) (ATR-Promotions: http://www.atr-p.com/face-db.html). For each expression (i.e., happy, fearful, or neutral), colour images of 4 male and 4 female models were displayed twice on the left or right. The facial stimuli were presented at vertical and horizontal visual angles of 9 and 6 degrees, respectively.

#### IBQ-R

The Infant Behavior Questionnaire-Revised (IBQ-R: Japanese version^26^) was used to assess the frequency of occurrence over the past 1 or 2 weeks of temperament-related behaviours on a 7-point scale ranging from *never* (1) to *always* (7). The IBQ-R includes 191 items on 14 scales. Previous factor analyses of IBQ-R scores identified three factor solutions: Positive Emotionality/Surgency, Negative Affectivity, and Orienting/Regulatory Capacity. Mothers were given the IBQ-R temperament questionnaire at the end of the session and were asked to complete and return it.

### Data analysis

Data with validity codes 0 and 1 for either eye were accepted (following the Tobii TX-300 user manual). Infant fixation confirmed that their gaze was recorded as focused on the area of the first central fixation stimulus (±132 pixel from the centre of the monitor) for more than 50% during 200 ms just before cue presentation. These fixated responses were defined as countable responses for the probability denominator. We define response time as elapsed time between peripheral cue (not target) presentation and the time at which the infant’s gaze passes the line indicating eye movement towards the target side because the cue presented for 300 ms may cause eye movement towards the cue’s location. The thresholds (i.e., the x-coordinate value used to detect eye movement towards the target stimulus) were set at 40% from the right and left edges, including a ~2.5-degree margin on both sides of the central fixation stimulus^36^, that is, 768 pixels from the left edge of the display for saccades to the left and 1152 pixels from the left edge of the display for saccades to the right. Additionally, any trials with a saccade commencing less than 100 ms or more than 2950 ms after peripheral cue onsets were not included. Moreover, a trained, experienced blind coder judged whether each trial was adequate for our analysis by referring to the videotape with frame-by-frame playback. That is, in both conditions, trials in which the infant’s gaze did not move directly from the central to the peripheral stimulus were not included. In the incongruent condition, trials were included in which the infant’s gaze initially moved from the central to the location of the facial cue directly, then from the cued location to the peripheral target directly. To establish reliability, infant gazing was coded by a second coder, who was also blind to the experimental conditions, for a random 20% of the infants. Cohen’s Kappa of interrater reliability was 0.89. Moreover, the latency of congruent or incongruent conditions for analysis was based on more than 2 trials per cell of 3 facial expressions. Thus, 16 infants were analysed longitudinally for the congruent condition, while in the incongruent condition, only 5 infants gave more than 2 trials per cell of 3 facial expressions at both 6 and 12 months. Although we did not analyse latencies in the incongruent condition or longitudinally, we did so for each age group.

With regard to temperament measured on the IBQ-R instrument, our study used scores for negative affectivity, effortful control, and a subscale of fear. With regard to attentional bias, we used latency in the congruent condition with a fearful face cue and response probability in the incongruent condition with a fearful face cue. In addition, we used the probability of infants’ initial shift gaze to face cues prior to the target on the opposite side.

## Data Availability

The authors declare that the data supporting the current findings are available from the corresponding author on reasonable request.
